# 14-Year Risk of All-Cause Mortality According to Hypoglycaemic Drug Exposure in a General Population

**DOI:** 10.1371/journal.pone.0095671

**Published:** 2014-04-21

**Authors:** Emilie Bérard, Vanina Bongard, Jean Dallongeville, Dominique Arveiler, Dominique Cottel, Aline Wagner, Jean-Bernard Ruidavets, Jean Ferrières

**Affiliations:** 1 Department of Epidemiology, Health Economics and Public Health, UMR-1027 INSERM, Toulouse University School of Medicine, Toulouse University Hospital, Toulouse, France; 2 Department of Epidemiology and Public Health, U-744 INSERM, Institut Pasteur de Lille, Université Lille Nord de France, Lille, France; 3 Department of Epidemiology and Public Health, EA 3430, Université de Strasbourg, Faculté de Médecine, Strasbourg, France; 4 Department of Cardiology B, Toulouse University Hospital, Toulouse, France; University of Texas Health Science Center at San Antonio, United States of America

## Abstract

**Purpose:**

Guidelines for management of patients with type 2 diabetes mellitus recommend the use of hypoglycaemic drugs when lifestyle interventions remain insufficient for glycaemic control. Recent trials have provided worrying safety data on certain hypoglycaemic drugs. The aim of this study was to assess 14-year risk of all-cause mortality according to hypoglycaemic drug exposure at baseline, in a general population.

**Methods:**

Our analysis was based on the observational Third French MONICA survey on cardiovascular risk factors (1995–1997). Vital status was obtained 14 years after inclusion, and assessment of determinants of mortality was based on multivariable Cox modelling.

**Results:**

There were 3336 participants and 248 deaths over the 14-year period. At baseline, there were 3162 (95%) non-diabetic, 46 (1%) untreated type 2 diabetic and 128 (4%) type 2 diabetic subjects with hypoglycaemic drug treatment (metformin alone (31%), sulfonylureas alone or in combination (49%), insulin alone or in combination (10%), or other treatments (9%)). After adjustment for duration of diabetes, history of diabetes complications, area of residence (centre), age, gender, educational level, alcohol consumption, smoking, blood pressure, LDL and HDL cholesterol, which all were significant and independent determinants of mortality, the hazard ratio for all-cause mortality was 3.22 [95% confidence interval: 0.87–11.9] for untreated diabetic subjects, 2.28 [0.98–5.26] for diabetics treated with metformin alone, 1.70 [0.92–3.16] for diabetics with sulfonylureas and 4.92 [1.70–14.3] for diabetic with insulin versus non-diabetic subjects.

**Conclusions:**

Our results support the conclusion that until more evidence is provided from randomized trials, a prudent approach should be to restrain use of insulin to situations in which combinations of non-insulin agents have failed to appropriately achieve glycemic control, as it is recommended in the current guidelines for the management of type 2 diabetes.

## Introduction

Guidelines for management of patients with type 2 diabetes mellitus recommend the use of hypoglycaemic drugs when lifestyle interventions remain insufficient for glycaemic control [1]. The recent ACCORD trial has provided worrying safety data on intensive treatment, reporting an early increased mortality compared with standard therapy [2]. On the other hand, only a few observational studies have evaluated in non-experimental conditions the long-term safety of hypoglycaemic treatments in the general population. Observational studies provide information that should be considered complementary to that provided by randomized clinical trials. The follow-up period is longer, whereas participants are usually followed for less than 5 years in clinical trials. Furthermore, observational studies provide data collected in a non-selected general population, while participants in clinical trials are generally under quite intensive clinical and biological management.

The aim of this study was to assess 14-year risk of all-cause mortality according to hypoglycaemic drug exposure (related to type 2 diabetes) at baseline in a non-experimental French general population.

## Materials and Methods

### Study population and design

A sample of 3403 subjects was recruited to participate in the Third French MONICA Survey on the prevalence of cardiovascular risk factors [3], [4]. Middle-aged men and women (35–64 years old), living in northern (Lille area), north-eastern (Strasbourg area) or south-western France (Toulouse area), were recruited between December 1994 and July 1997. Polling lists available in each town hall of the survey areas were used to obtain the stratified random sample. Stratification was applied according to centre, town size (rural versus urban), age and gender, in order to obtain 200 subjects in each 10-year age group (35–44, 45–54 and 55–64 years), gender and centre. No incentive to participate (in particular no financial incentive) was offered. Written informed consent to study participation was obtained from each subject after full explanation of the nature of the research. The participation rate was 66% [4].

Vital status on December 31, 2009 was obtained for each participant through the national database that records each year all deaths occurring in the French population (RNIPP) [5]. Authorizations to use these data were obtained in accordance with French law (Commission nationale de l'informatique et des libertés (CNIL): authorization 355152v1, September 3, 2008).

### Ethics statement

The study protocol was approved by an institutional ethics committee, the Comité Consultatif de Protection des Personnes dans la Recherche Biomédicale (CCPPRB), in Lille, France, on January 19, 1995 (CP 95/04) in accordance with French law on human biomedical research and the Declaration of Helsinki.

### Questionnaires and measurement of clinical parameters

At baseline, extensive questionnaires were filled in by trained medical staff during a face-to-face interview with the participant. Information on exposures was collected at baseline only. Data concerning socio-economic level, personal medical history, cardiovascular risk factors, lifestyle habits and drug intake were recorded. Participants were asked to bring their latest drug prescription at the inclusion visit. All drugs taken during the two weeks preceding the visit were recorded (international nonproprietary name (INN) and dosage). Hypoglycaemic drug use was assessed as the current consumption of a drug prescribed by a physician for treatment of glycaemic disturbances. Only metformin, sulfonylureas and insulin were considered as hypoglycaemic drugs. Education level was assessed by a report of graduation or level of school drop-out. People who currently smoked or who had stopped for less than 3 years were considered as smokers. Alcohol consumption was quantified in grams of alcohol per day by 7-day recall of a typical week. Four levels of leisure time physical activity were defined: no regular physical activity, light physical activity (such as walking or bicycling, without causing shortness of breath or sweating, almost every week), moderate physical activity (i.e. causing shortness of breath or sweating, during at least 20 minutes, once or twice a week) and high physical activity (i.e. causing shortness of breath or sweating, during at least 20 minutes, three times a week or more). Height, weight and arterial blood pressure (mean of two measurements performed with a standard sphygmomanometer in a sitting position after at least 5 minutes rest) were measured according to standardised protocols by the medical staff. Hypertension was assessed for people with blood pressure ≥160/95 mmHg according to the threshold recommended for measurements performed at a single visit at the time of recruitment (1994–1997) [6] in order to limit false positives. Body mass index was calculated as weight divided by height squared (kg/m^2^).

### Laboratory methods

Blood samples were taken after at least 10 hours of overnight fasting. Serum total cholesterol and triglycerides were measured by enzymatic assays (Boehringer, Mannheim, Germany). High density lipoprotein cholesterol (HDL cholesterol) measurement was done after sodium phosphotungstate-magnesium chloride precipitation of apo B-containing lipoproteins. Low density lipoprotein cholesterol (LDL cholesterol) was determined by the Friedewald formula when triglycerides were below 4.6 mmol/L (400 mg/dL) [7]. Glucose levels were measured using a conventional enzymatic method based on hexokinase-glucose-6-phosphate deshydrogenase. Diabetes was assessed for subjects receiving hypoglycaemic drugs or with fasting blood glucose ≥7.7 mmol/L (140 mg/dL) according to the threshold that was currently used in clinical practice at baseline (1994–1997) [8]. Since only three subjects in our sample were likely to present with a diagnosis of type 1 diabetes (subjects solely treated with insulin at baseline who had been diagnosed with diabetes before the age of 25), we restrained our analysis to type 2 diabetic subjects, only.

### Statistical analysis

Statistical analysis was performed on STATA statistical software, release 11.2 (STATA Corporation, College Station, TX, USA).

Subjects with the following medical histories were excluded from the analysis: chronic renal failure (International Classification of Disease, 9th revision, codes 585.0 to 585.9), chronic respiratory insufficiency (496.0 to 496.9), chronic heart failure (428.0 to 428.9), chronic liver disease or cirrhosis (571.0 to 571.9) and cancer, excluding benign neoplasms and in-situ carcinoma (140.0 to 209.9 and 235.0 to 239.9). Overall, 64 participants were excluded from the analysis using these criteria.

We first described baseline characteristics of participants and compared baseline characteristics by outcome occurrence, comparing subjects who did not die (i.e. those alive on December 31, 2009) with subjects who had a fatal event during follow-up. Qualitative variables were compared between groups using the χ^2^-test (or Fisher's exact test when necessary). Student's *t*-test was used to compare the distribution of quantitative data (or Mann-Whitney's test when distribution departed from normality or when homoscedasticity was rejected).

Survival analysis was then conducted. Events were cases of death and exposure was defined by the treatment with hypoglycaemic drugs at inclusion. Hazard ratios (HRs) for mortality and 95% confidence intervals (CI) were assessed using a Cox model. The independent variables initially introduced in the survival model were hypoglycaemic drug treatments and all variables associated with mortality in univariate analysis with a p-value<0.20. A backward analysis was then applied until only variables significantly and independently associated with mortality (p-value<0.05) remained. Since the log-linearity hypothesis was not fully respected, the following continuous variables were transformed into ordered data: age (35–44, 45–54 and 55–64 years), alcohol consumption (<80 g/day and ≥80 g/day), blood pressure (<160/95 mmHg and ≥160/95 mmHg), LDL cholesterol (<5.2 mmol/L (200 mg/dL) and ≥5.2 mmol/L), HDL cholesterol (<0.80 mmol/L (30 mg/dL) for men or <1.30 mmol/L (50 mg/dL) for women and ≥0.80 mmol/L for men or ≥1.30 mmol/L for women) and duration of diabetes (<6 years, ≥6 years and non-diabetic subjects). The proportional-hazard assumption was tested for each covariate by the “log-log” plot method curves ((-ln{-ln(survival)}), for each category of nominal covariate, versus ln(analysis time)). None of the assumptions could be rejected.

## Results

### Description of the population sample

**Table 1** describes the main baseline characteristics of participants. There were 3336 subjects equally distributed by centre and gender and the mean age was 50 years (±8 years). This sample included 1% (n = 46) of untreated type 2 diabetic subjects (subjects with fasting blood glucose >7.7 mmol/L (140 mg/dL) and untreated) and 4% (n = 128) of type 2 diabetics with hypoglycaemic drug treatment including 31% (n = 40) with metformin alone, 49% (n = 63) with sulfonylureas alone or in combination with other hypoglycaemic drugs except insulin, 10% (n = 13) with insulin alone or in combination with other hypoglycaemic drugs except sulfonylureas, and 9% (n = 12) with other treatments. Of subjects treated with sulfonylureas, 62% (n = 39) were treated with gliclazide, 29% (n = 18) with glibenclamide, and 9% (n = 6) with other sulfonylureas. Of subjects treated with insulin, 38% (n = 5) were treated with intermediate-acting insulin (duration between 8 and 20 h) associated or not with metformin, 38% (n = 5) with intermediate or long-acting insulin (duration >20 h) associated with short-acting insulin (duration <8 h), 8% (n = 1) with short-acting insulin alone, and 15% (n = 2) with insulin (duration unknown) associated or not with metformin.

**Table 1:** Main baseline characteristics of participants.

	Total N = 3336	Non-diabetics£ N = 3162	Untreated diabetics N = 46	Diabetics with metformin££ N = 40	Diabetics with sulfonylureas£££ N = 63	Diabetics with insulin£££ N = 13
Lille, N (%)	1107 (33.2)	1037 (32.8)	20 (43.5)	22 (55.0)	17 (27.0)	6 (46.1)
Strasbourg, N (%)	1058 (31.7)	1004 (31.8)	13 (28.3)	13 (32.5)	21 (33.3)	5 (38.5)
Toulouse, N (%)	1171 (35.1)	1121 (35.4)	13 (28.3)	5 (12.5)	25 (39.7)	2 (15.4)
Age (years), mean (± SD)	50.4 (±8.3)	50.1 (±8.3)	53.6 (±7.7)	56.2 (±6.6)	56.5 (±7.7)	55.0 (±8.5)
Men, N (%)	1683 (50.5)	1585 (50.1)	27 (58.7)	26 (65.0)	38 (60.3)	4 (30.8)
Educational level < high school completion, N (%)	2221 (66.6)	2076 (65.7)	35 (76.1)	34 (85.0)	53 (84.1)	12 (92.3)
Smoking, N (%)	989 (29.7)	947 (30.0)	11 (23.9)	8 (20.0)	17 (27.0)	3 (23.1)
Drinking >80 g/d, N (%)	124 (3.7)	109 (3.5)	6 (13.0)	3 (7.5)	6 (9.5)	0 (0.0)
No physical activity, N (%)	740 (22.2)	694 (22.0)	11 (23.9)	11 (27.5)	17 (27.0)	4 (30.8)
Low physical activity almost every week, N (%)	1637 (49.2)	1545 (48.9)	26 (56.5)	23 (57.5)	28 (44.4)	8 (61.5)
Intense physical activity (at least 20 minutes 1 to 2 times a week), N (%)	581 (17.4)	558 (17.7)	7 (15.2)	4 (10.0)	10 (15.9)	1 (7.7)
Intense physical activity (at least 20 minutes 3 times a week or more), N (%)	373 (11.2)	360 (11.4)	2 (4.4)	2 (5.0)	8 (12.7)	0 (0.0)
Body mass index (kg/m^2^), mean (± SD)	26.3 (±4.6)	26.1 (±4.5)	30.9 (±4.9)	31.3 (±7.0)	29.4 (±5.7)	30.5 (±6.9)
Systolic blood pressure (mmHg), mean (± SD)	132 (±19)	132 (±19)	144 (±17)	144 (±19)	145 (±22)	143 (±22)
Diastolic blood pressure (mmHg), mean (± SD)	83 (±12)	82 (±12)	88 (±12)	88 (±12)	86 (±11)	82 (±14)
LDL cholesterol (mmol/L), mean (± SD)	3.84 (±0.99)	3.85 (±0.99)	4.10 (±1.07)	3.41 (±0.75)	3.74 (±0.94)	3.11 (±1.38)
HDL cholesterol (mmol/L), mean (± SD)	1.48 (±0.44)	1.49 (±0.44)	1.20 (±0.49)	1.10 (±0.30)	1.25 (±0.31)	1.66 (±0.62)
Triglycerides (mmol/L), mean (± SD)	1.32 (±1.36)	1.25 (±0.99)	3.12 (±5.77)	3.08 (±5.23)	2.01 (±1.56)	1.18 (±0.49)
History of diabetes complications* ^,^ **, N (%)	17 (9.8)		0 (0.0)	8 (20.0)	7 (11.1)	2 (15.4)
Duration of diabetes (years)**, median (IQR)	7 (2–13)		2 (1–10)	5 (2–10)	10 (4–16)	12 (7–19)

Of type 2 diabetic subjects, 12 were receiving other hypoglycaemic treatments. SD: Standard Deviation; IQR: Inter-Quartile Range;

£ Diabetes was assessed for subjects with fasting blood glucose ≥7.7 mmol/l (140 mg/dl) or under hypoglycaemic drug treatment;

££ Diabetic subjects treated with metformin alone;

£££ Diabetics with sulfonylureas or insulin alone or in combination;

* Diabetes with renal, ophthalmic, neurological, peripheral arterial disease or atherosclerotic cardiovascular disease;

** Among diabetics.

During the 14-year follow-up, 248 deaths were recorded. The death rate was 7% [95% confidence interval 6%–8%] in subjects without diabetes, 20% [9%–34%] in untreated type 2 diabetic subjects and 21% [14%–29%] in type 2 diabetics treated with hypoglycaemic drugs (23% [11%–38%] in diabetics with metformin alone, 19% [10%–31%] in diabetics treated with sulfonylureas (alone or in combination) and 31% [9%–61%] in diabetics treated with insulin (alone or in combination). The death rate was not significantly different for the various classes of sulfonylureas or insulin.

**Table 2** describes the main baseline characteristics of deceased and non-deceased participants. Area of residence (centre), age, male gender, low educational level, smoking, drinking (>80 g/d), low physical activity, high body mass index, high blood pressure or LDL cholesterol, low HDL cholesterol and high triglycerides were significantly associated with death. As expected, type 2 diabetes was also significantly associated with death. Regardless of use of hypoglycaemic treatment, type 2 diabetic subjects were more numerous in the group of deceased subjects than in the group of non-deceased subjects. Moreover, a history of complications related to type 2 diabetes such as renal failure, ophthalmic complications, neuropathy, coronary or peripheral arterial disease was also significantly associated with death.

**Table 2:** Main baseline characteristics of participants according to death occurrence.

	Non deceased N = 3088	Deceased N = 248	*p*-value
Lille, N (%)	993 (32.2)	114 (46.0)	<0.001
Strasbourg, N (%)	993 (32.2)	65 (26.2)	
Toulouse, N (%)	1102 (35.7)	69 (27.8)	
Age (years), mean (± SD)	50.0 (±8.2)	55.5 (±7.7)	<0.001
Men, N (%)	1507 (48.8)	176 (71.0)	<0.001
Educational level < high school completion, N (%)	2020 (65.4)	201 (81.1)	<0.001
Smoking, N (%)	867 (28.1)	122 (49.2)	<0.001
Drinking >80 g/d, N (%)	96 (3.1)	28 (11.3)	<0.001
No physical activity, N (%)	675 (21.9)	65 (26.2)	0.017
Low physical activity almost every week, N (%)	1512 (49.0)	125 (50.4)	
Intense physical activity during at least 20 minutes 1 to 2 times a week, N (%)	555 (18.0)	26 (10.5)	
Intense physical activity during at least 20 minutes 3 times a week or more, N (%)	341 (11.1)	32 (12.9)	
Body mass index (kg/m^2^), mean (± SD)	26.2 (±4.6)	27.3 (±5.5)	0.002
Systolic blood pressure (mmHg), mean (± SD)	132 (±19)	141 (±21)	<0.001
Diastolic blood pressure (mmHg), mean (± SD)	82 (±12)	85 (±13)	0.001
LDL cholesterol (mmol/L), mean (± SD)	3.84 (±0.98)	3.86 (±1.14)	0.822
LDL cholesterol ≥5.2 mmol/L (200 mg/dl), N (%)	239 (8.0)	32 (13.9)	0.002
HDL cholesterol (mmol/L), mean (± SD)	1.48 (±0.44)	1.39 (±0.46)	<0.001
Triglycerides (mmol/L), mean (± SD)	1.29 (±1.36)	1.66 (±1.40)	<0.001
Non-diabetic subjects*, N (%)	2949 (95.5)	213 (85.9)	<0.001
Untreated diabetics, N (%)	37 (1.2)	9 (3.6)	
Diabetics with metformin alone, N (%)	31 (1.0)	9 (3.6)	
Diabetics with sulfonylureas (alone or in combination), N (%)	51 (1.7)	12 (4.8)	
Diabetics with insulin (alone or in combination), N (%)	9 (0.3)	4 (1.6)	
History of diabetes complications** ^,^ ***, N (%)	8 (5.8)	9 (25.7)	0.002
Duration of diabetes (years)***, median (IQR)	6 (2–12)	10 (5–16)	0.101

SD: Standard Deviation; IQR: Inter-Quartile Range;

* Diabetes was assessed for subjects with fasting blood glucose ≥7.7 mmol/l (140 mg/dl) or under hypoglycaemic drug treatment;

** Diabetes with renal, ophthalmic, neurological, peripheral arterial disease or atherosclerotic cardiovascular disease;

*** Among diabetic subjects.

### Survival analysis

**Table 3** shows the adjusted 14-year risk of all-cause mortality in type 2 diabetic compared with non-diabetic subjects. After adjustment for duration of diabetes, history of diabetes complications (diabetes with renal, ophthalmic, neurological, peripheral arterial disease or atherosclerotic cardiovascular disease), area of residence (centre), age, gender, educational level, alcohol consumption, smoking, blood pressure, LDL and HDL cholesterol, which all were significant and independent determinants of mortality, the adjusted hazard ratio for all-cause mortality was 3.22 [0.87–11.9] (p = 0.080) for untreated diabetic subjects, 2.28 [0.98–5.26] (p = 0.055) for diabetics treated with metformin alone, 1.70 [0.92–3.16] (p = 0.092) for diabetics with sulfonylureas (alone or in combination) and 4.92 [1.70–14.3] (p = 0.003) for diabetic subjects treated with insulin (alone or in combination) compared with non-diabetic subjects. Using the same model combining diabetic subjects treated with metformin alone or with sulfonylureas (alone or in combination), adjusted HR was 1.85 [1.07–3.19] (p = 0.027) for diabetic subjects with non-insulin agents versus non-diabetics.

**Table 3:** Adjusted HR for mortality in type 2 diabetic compared with non-diabetic subjects.

	HR	95% CI	*p*-value
**Untreated diabetic subjects (1)**	**3.22**	**[0.87–11.9]**	**0.080**
**Diabetics with metformin alone (1)** £	**2.28**	**[0.98–5.26]**	**0.055**
**Diabetics with sulfonylureas (alone or in combination) (1)** £	**1.70**	**[0.92–3.16]**	**0.092**
**Diabetics with insulin (alone or in combination) (1)**	**4.92**	**[1.70–14.3]**	**0.003**
Duration of diabetes ≥6 years*	0.68	[0.28–1.62]	0.379
**History of diabetes complications** **	**1.73**	**[1.13–2.67]**	**0.012**
Strasbourg (2)	1.09	[0.76–1.55]	0.645
Lille (2)	1.73	[1.27–2.36]	0.001
**45–54 years (3)**	**1.72**	**[1.15–2.58]**	**0.008**
**55–64 years (3)**	**4.35**	**[2.98–6.36]**	**<0.001**
Men	2.14	[1.56–2.94]	<0.001
**Educational level < high school completion**	**1.60**	**[1.15–2.21]**	**0.005**
Smoking	2.57	[1.97–3.36]	<0.001
**Drinking >80 g/d**	**1.88**	**[1.23–2.87]**	**0.003**
Blood pressure ≥160/95 mmHg	1.41	[1.06–1.88]	0.018
**LDL cholesterol ≥5.2 mmol/L (200 mg/dl)**	**1.54**	**[1.05–2.27]**	**0.027**
HDL cholesterol <0.8 mmol/L (30 mg/dl) for men or <1.3 mmol/l (50 mg/dl) for women	1.60	[1.09–2.35]	0.016

HR: Hazard Ratio; CI: Confidence Interval; (1) Reference: Non-diabetic subjects; (2) Reference: Toulouse; (3) Reference: 35–44 years.

£ HR for type 2 diabetics with metformin alone or with sulfonylureas (alone or in combination) = 1.85 [1.07–3.20] (p = 0.027);

* As compared with <6 years duration of diabetes (among diabetics). Duration of diabetes is composed of 3 categories (<6 years, ≥6 years and non-diabetic subjects);

** Diabetes with renal, ophthalmic, neurological, peripheral arterial disease or atherosclerotic cardiovascular disease.

## Discussion

In this non-experimental study carried out in the general population, we showed that after adjustment for severity of type 2 diabetes at baseline (duration of the disease and history of micro- or macrovascular complications) and presence of major risk factors for mortality (area of residence, age, gender, educational level, drinking, smoking, hypertension, LDL and HDL cholesterol), type 2 diabetic subjects treated with insulin had a mortality risk that was about 5 times higher than non-diabetic subjects (adjusted HR = 4.92 [1.70–14.3], p = 0.003).

Several explanations and hypotheses can be put forward. The first is based on the non-experimental design of our study. Despite extensive adjustment for severity of diabetes at baseline and presence of major mortality risk factors, the risk of death observed in diabetic subjects treated with insulin may be due to an indication bias, i.e. may be related to differences in the severity of diabetes, with cases probably more severe in subjects treated with insulin than in those treated with non-insulin agents (metformin or sulfonylureas). We could not adjust our analysis for HbA1c (which reflects glycaemic balance during the last 3 months) because our work was based on data collected during the Third French MONICA Survey which was initially designed to estimate the prevalence of cardiovascular risk factors. There was no indication to include HbA1c in this protocol, as this measure is only recommended in monitoring and not in screening for diabetes. However, at baseline, mean fasting blood glucose was 10 mmol/L in untreated type 2 diabetic subjects, 9 mmol/L in type 2 diabetics treated with metformin alone or with sulfonylureas, and 11 mmol/L in type 2 diabetics treated with insulin, which is very close. In the same way, screening for microalbuminuria (an independent cardiovascular risk factor in type 2 diabetes) was not included in the protocol, which could lead to insufficient adjustment for the severity of diabetes. However, the adjustment for major mortality risk factors seems appropriate. Apart from age, the main risk factors for cancers are smoking and alcohol consumption, and these were extensively recorded in our study together with cardiovascular risk factors. We also took into account global markers of health condition (educational level and area of residence) known to be strongly associated with life expectancy [9], [10]. We therefore believe that our adjustment for major mortality risk factors is appropriate.

On the other hand, other studies (experimental and observational) in different populations had studied the risk associated with hypoglycaemic treatment and their results are in line with ours. Our assumption concerning the higher risk of death for type 2 diabetic subjects treated with insulin therefore considers the possibility that insulin itself may have deleterious effect. It is widely accepted that insulin promotes severe hypoglycaemia and weight gain [1]. For this reason, insulin is indicated only from second step in the management of type 2 diabetes, when the risk-benefit ratio becomes acceptable. Current guidelines [1] on the management of type 2 diabetic patients consider that the first-step treatment should be based on lifestyle interventions and metformin. If first-step treatment fails (does not achieve/maintain an HbA1c target over ∼3 months), a second non-insulin agent is introduced (a sulfonylurea or thiazolidinedione or DPP-4 inhibitor or GLP-1 receptor agonist) or basal insulin. In the third-step treatment, two non-insulin agents were combined with a third non-insulin agents or basal insulin. It is also in the fourth-step treatment, that intensive insulin therapy (basal insulin and short- or rapid-acting insulin) is recommended in combination with one or two non-insulin agents. Intensive treatment of type 2 diabetes, usually based on insulin, has not so far demonstrated a positive risk-benefit ratio. A recent meta-analysis [11] compiling trials that compared intensive management (using insulin) with standard treatment found a small benefit in the intensive therapy group concerning non-fatal myocardial infarction and microalbuminuria, which was offset by a significant risk of severe hypoglycaemia. These results led the authors to the conclusion that further trials appear necessary before advising intensive management of diabetic patients. In addition, two recent North American cohorts have shown that diabetic subjects treated with insulin compared with those not treated with insulin are at higher risk of myocardial infarction [12] and mortality (cardiovascular, non-cardiovascular and all-cause mortality) [13]. Insulin may have a deleterious cardiovascular effect by stimulating the sympathetic nervous system involving vasoconstriction and thus promoting atherosclerosis [14]. On the other hand, insulin is known to promote proliferation and resistance to apoptosis of cancer cells [15], [16]. In our study, half the deaths among type 2 diabetic subjects treated with insulin were due to a cardiovascular cause and one quarter was due to a cancer. Moreover, the risk of cancer disease and mortality is higher in patients treated with insulin [17], [18], [19]. Unfortunately, to the best of our knowledge, there is no clinical trial comparing the efficacy and safety of insulin with other hypoglycaemic treatments, partly because they are often combined.

This study has further limitations that must be addressed. First, results are based on small numbers of deaths in type 2 diabetic subjects (9, 9, 12 and 4 deaths in untreated diabetics, diabetics treated with metformin alone, with sulfonylureas and with insulin, respectively). Despite the small number of deaths in type 2 diabetic patients treated with insulin, the increased risk of death in these subjects is important and highly significant, which is why we have considered it worthwhile. In fact 217 deaths (213 in non-diabetic subjects and 4 in insulin-treated type 2 diabetic subjects) provide a power greater than 80% to detect a HR≥1.5 with two sided type 1 error rate of 5% (α = 0.05) for the comparison of two exponential survival distributions [20]. Another limitation is related to possible changes in exposure to hypoglycaemic therapy during follow-up. With the ageing of the population, it is likely that new cases of diabetes appeared over the 14-year follow-up, leading to initiation of hypoglycaemic therapy. Accordingly, and given that exposure could not be collected at the end of the study, subjects may have been misclassified regarding their exposure to a glucose-lowering therapy.

Despite these limitations, we believe our small albeit long-term study provides information that should be considered complementary to that provided by randomized clinical trials. The follow-up period lasted 14 years, whereas participants are usually followed for less than 5 years in clinical trials. Furthermore, we provide data collected in a non-selected general population, while participants in clinical trials are generally under quite intensive clinical and biological management.

## Conclusion

In this non-experimental study, after extensive adjustment for severity of diabetes and mortality risk factors, diabetic patients treated with insulin were at increased risk for 14-year all-cause mortality. However, owing to the observational design of the study, this does not draw a causal link between use of insulin and risk of mortality. It is indeed possible that some form of bias or residual confounding may provide an alternate explanation for our findings. Randomization is the only method allowing appropriate control of confounding bias, and our data are non-randomized. Still, the lack of proven clinical benefit regarding the use of insulin among type 2 diabetic patients in recent clinical trials is concerning. Therefore, we believe that our results support the conclusion that until more evidence is provided from randomized trials, a prudent approach should be to restrain use of insulin to situations in which combinations of non-insulin agents have failed to appropriately achieve glycemic control, as it is recommended in the current guidelines for the management of type 2 diabetes [1].
